# Peanut applied to the skin of nonhuman primates induces antigen‐specific IgG but not IgE

**DOI:** 10.1002/iid3.296

**Published:** 2020-03-27

**Authors:** Michael D. Kulis, Johanna M. Smeekens, Kylie Kavanagh, Matthew J. Jorgensen

**Affiliations:** ^1^ Division of Allergy, Immunology, and Rheumatology, Department of Pediatrics University of North Carolina Chapel Hill North Carolina; ^2^ Department of Pediatrics, University of North Carolina Food Allergy Initiative University of North Carolina Chapel Hill North Carolina; ^3^ Department of Pathology, Section on Comparative Medicine Wake Forest School of Medicine Winston‐Salem North Carolina; ^4^ Department of Biomedicine University of Tasmania Hobart Tasmania Australia

**Keywords:** African green monkey, cutaneous, food allergy, IgE, IgG, nonhuman primate, peanut allergy

## Abstract

**Introduction:**

Previous studies in humans support the dual‐allergen exposure hypothesis, and several studies in mouse models have demonstrated that cutaneous exposure to disrupted or intact skin can lead to sensitization to peanut. However, the field lacks definitive evidence that cutaneous exposure leads to peanut allergy in humans or other primates.

**Methods:**

Peanut extract was applied to the shaved back of the neck of four male and four female African green monkeys three times per week for 4 weeks. An oral food challenge (OFC) was performed the following week by gavage of 200 mg of peanut protein, and vital signs were monitored for 30 minutes post‐OFC. Blood was collected at baseline, day 11, day 32, and 30 minutes post‐OFC. Total IgE, and peanut‐specific immunoglobulin E (IgE) and immunoglobulin G (IgG) were quantified in serum collected throughout the 4 weeks. Histamine was measured in serum collected 30 minutes post‐OFC.

**Results:**

Peanut‐specific IgE was undetectable at any time points in any of the monkeys, and there was no consistent increase in total IgE. During the oral challenge, none of the monkeys experienced allergic symptoms and histamine levels did not change. However, seven of the eight monkeys produced increasing peanut‐specific IgG by day 32, indicating that repeated skin exposure to peanut is immunogenic.

**Conclusions:**

Skin exposure to peanut did not lead to sensitization in this study, and monkeys did not experience anaphylaxis upon peanut challenge. However, monkeys produced increased peanut‐specific IgG throughout peanut exposure, indicating that repeated skin exposure to peanut is immunogenic.

## Graphical abstract

Peanut proteins were applied to the skin of African green monkeys thrice per week for four weeks. Peanut‐specific IgE was not detected in serum throughout peanut exposure, and monkeys did not experience anaphylaxis upon peanut challenge, indicating that monkeys were not sensitized to peanut. However, peanut‐specific IgG was increased in seven of eight monkeys over the exposure period, indicating that repeated skin exposure to peanut is immunogenic.

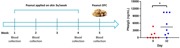

## 1 INTRODUCTION

Peanut allergy affects an estimated 1% of children in the United States and the prevalence has increased over the past two decades.^1^ A long‐standing observation by allergists is that children diagnosed with a peanut allergy react on their first known ingestion, implying that sensitization to peanut antigens may have occurred through a nonoral route. There is mounting evidence supporting the role of allergen exposure through the skin and subsequent development of peanut allergy. For example, a strong risk factor for peanut allergy is severe eczema and filaggrin mutations that lead to disrupted skin barrier function.^2^, ^3^ In addition, peanut allergens found in house dust samples contain biologically active allergens that can cross‐link immunoglobulin E (IgE) on basophils indicating an environmental source of allergens.^4^ The concept of skin barrier impairment and environmental exposure to peanut antigens has been investigated in the United States and the United Kingdom with results indicating associations of increased risk in children with disrupted skin barrier and dose‐dependent exposure to peanut as estimated by quantities of allergen in the house dust.^5^, ^6^

Another important observation connecting early dietary introduction of peanut in Israel vs delayed introduction in the United Kingdom and peanut allergy development was made approximately 10 years ago. Researchers found a 10‐fold increased risk for having peanut allergy in the UK population and attributed the decreased prevalence in Israel to early oral exposure to peanut.^7^ These researchers have since developed the dual‐allergen exposure hypothesis, which theorizes that tolerance is induced through oral exposure to high quantities of antigen, and sensitization and subsequent allergy is induced through cutaneous exposure to low quantities of antigen, before oral exposure during infancy.^6^ Specifically, genetically predisposed infants that are first cutaneously exposed to peanut, before oral consumption, are more likely to develop an allergy. A landmark clinical trial titled Learning Early About Peanut Allergy (LEAP) demonstrated that exposing infants less than 1 year of age to peanut orally several times per week significantly reduced the likelihood of developing an allergy later in childhood, compared to infants who were not orally ingesting peanuts.^8^ While all of these studies support the dual‐allergen exposure hypothesis, the field lacks definitive evidence that cutaneous exposure leads to peanut allergy in humans or other primates.

Several studies in mouse models have demonstrated that cutaneous exposure to disrupted or intact skin can lead to sensitization to various foods, including peanut.^9^, ^10^, ^11^, ^12^, ^13^ A particularly interesting study demonstrated that peanut itself has adjuvant properties and can induce sensitization to other food antigens simultaneously applied to the rodent's skin.^13^ In that study, 1 mg of peanut extract in 50 to 100 µL was applied on the skin and left to dry. Cutaneous exposure was repeated once weekly for 6 weeks, then an oral peanut challenge was performed 1 week later. We aimed to determine whether a similar protocol would lead to sensitization (ie, peanut‐specific IgE in serum) and allergy (ie, anaphylaxis upon oral peanut challenge) in a nonhuman primate model.

## 2 METHODS

**Figure 1:**
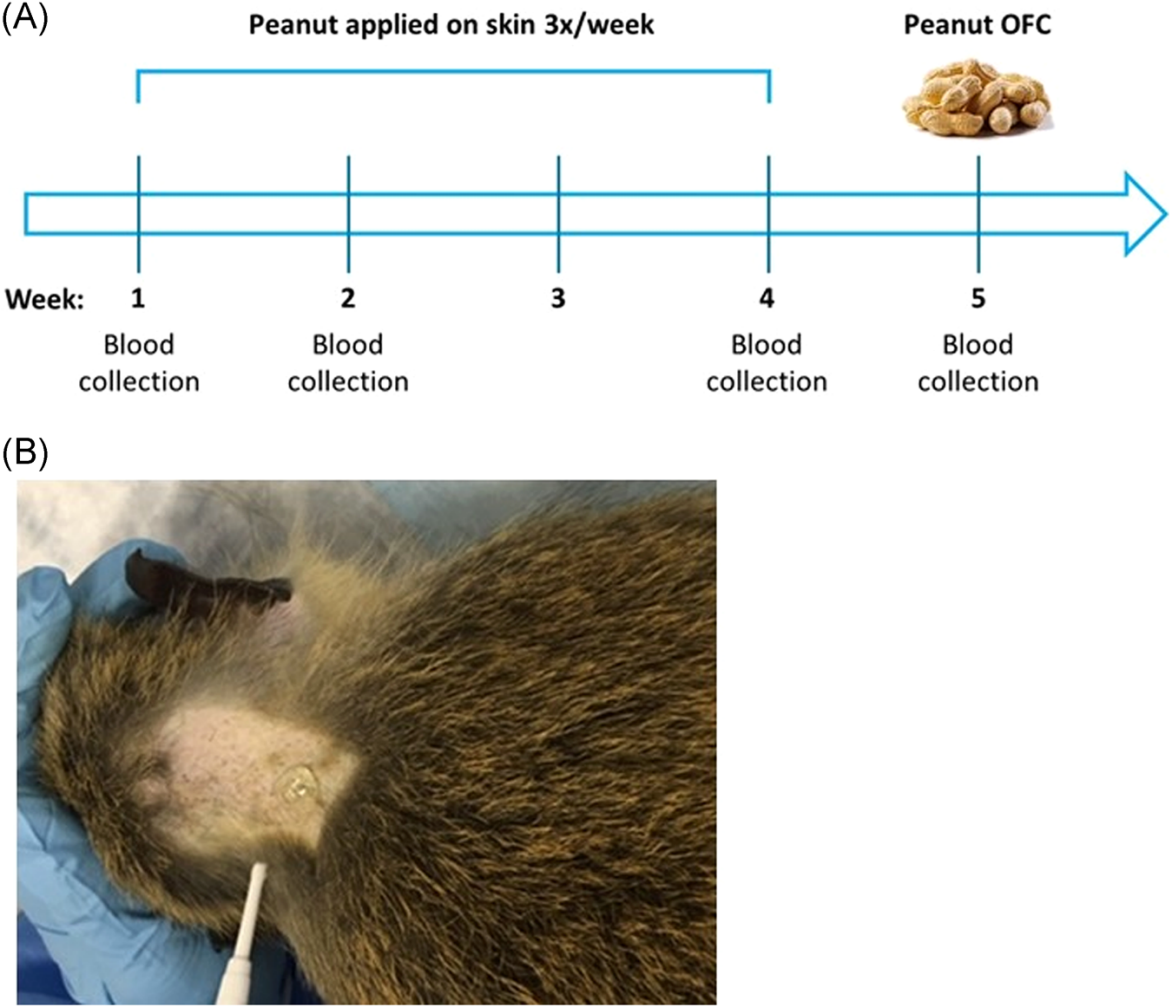
Study design. A, Experimental scheme indicating peanut exposure regimen, blood collection, and oral food challenge (OFC). B, Photo of a monkey with peanut extract applied on the shaved area on the back of the neck

Our study population was 1.2‐ to 1.4‐year‐old African green monkeys (*Chlorocebus aethiops sabaeus*) housed at the Wake Forest School of Medicine that had never ingested peanut as part of their diet. Eight monkeys were enrolled in the study, four females and four males. The study design is depicted in Figure 1A; the protocol was approved by the Wake Forest IACUC (protocol # A18‐138) and complied with the ARRIVE guidelines. A small area on the back of the neck was shaved using electric clippers for application of peanut antigens (Figure 1B), to prevent monkeys from licking or rubbing the application site. Peanut extract containing all major allergens was made from a defatted, lightly roasted peanut flour (Golden Peanut) extracted in phosphate‐buffered saline, as previously described.^14^ Glycerol was added to the aqueous peanut extract to provide viscosity and prevent immediate runoff from the skin. One milligram of peanut in 200 µL was applied, and left to dry, three times per week (Monday, Wednesday, and Friday) for 4 weeks. Blood was collected by femoral venipuncture at baseline, day 11, and day 32 under ketamine anesthesia (10 mg/kg, intramuscular). An oral food challenge (OFC) was performed, also under ketamine anesthesia, the following week by gavage of 200 mg of peanut extract (10 mL of a 20 mg/mL solution) and a blood sample was collected 30 minutes post‐OFC. Vital signs, including respiration rate, heart rate, blood pressure, partial pressure of blood oxygenation, rectal temperature, and observable skin reactions, were monitored for 30 minutes post‐OFC.

Serum was assessed for both total IgE and peanut‐specific IgE by enzyme‐linked immunosorbent assay (ELISA). Total IgE was quantified using a kit from Life Diagnostics whereas peanut‐specific IgE was quantified with the peanut extract used for skin applications and the anti‐monkey IgE from the Life Diagnostics kit. Peanut‐specific immunoglobulin G (IgG) was measured in serum collected throughout the course of peanut exposure via ELISA, using peanut extract to coat plates and an anti‐monkey IgG antibody for detection (Abcam). Histamine levels were measured in serum collected 30 minutes post‐OFC by ELISA (Beckman Coulter).

## 3 RESULTS

**Figure 2:**
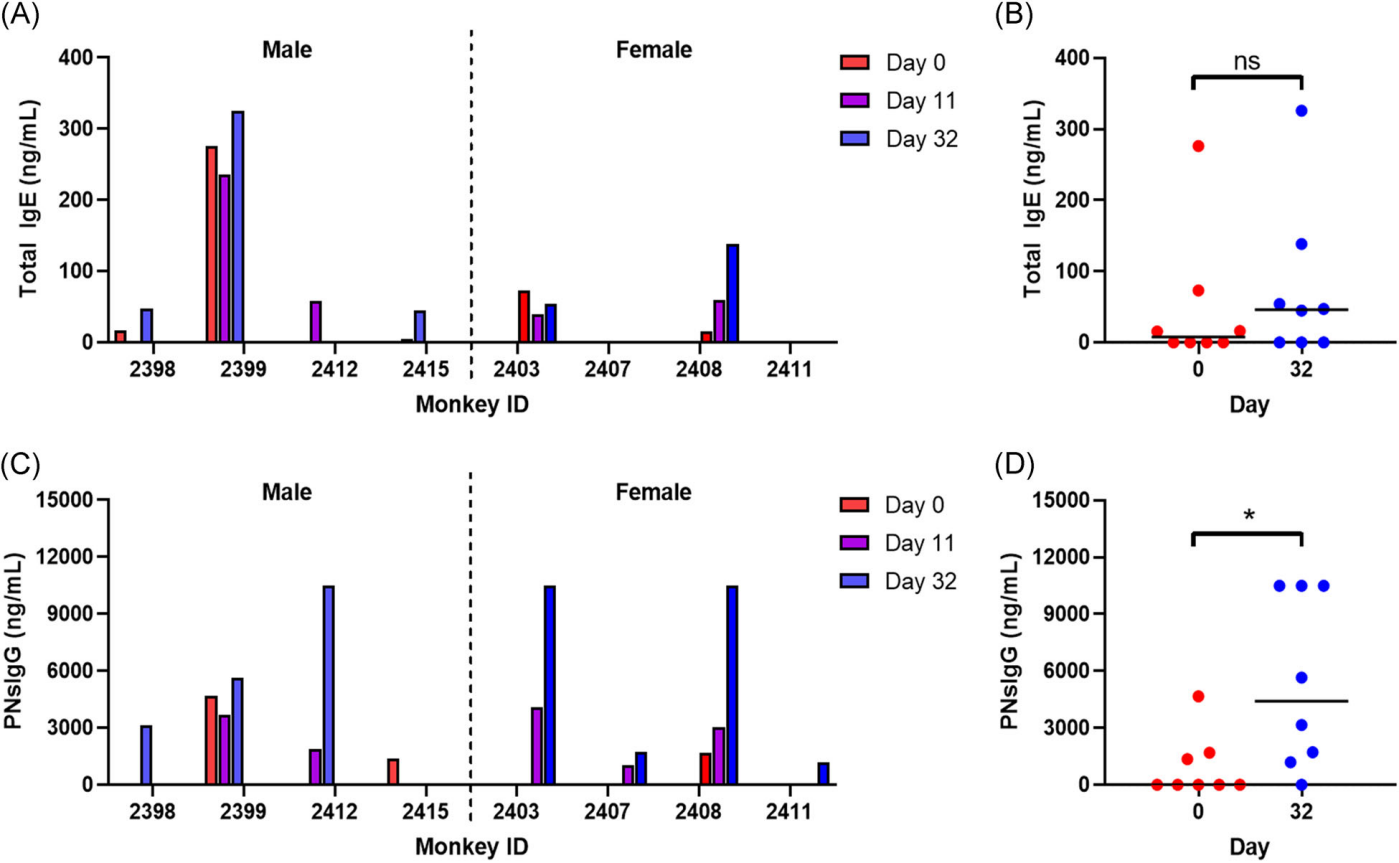
Immunoglobulin production throughout the course of peanut exposure. A, Total IgE in the serum of individual male and female monkeys. B, Grouped IgE data at days 0 and 32. C, Peanut‐specific IgG in the serum of individual male and female monkeys. D. Grouped IgG data at days 0 and 32. IgE, immunoglobulin E; IgG, immunoglobulin G. *P < .05

To determine whether the monkeys became sensitized to peanut, serum was assessed for both total IgE and peanut‐specific IgE by ELISA. Some monkeys had increased IgE during the peanut exposure period; however, this was not consistent across animals and did not reach statistical significance (Figure 2A,B). We were unable to detect peanut‐specific IgE, even at a 1:2 dilution, at any time points in any of the monkeys. During the oral challenge, none of the monkeys experienced anaphylaxis as evidenced by a lack of allergic symptoms and changes in vitals. Histamine levels did not change relative to their day 32 levels and were not different than naïve monkeys that were challenged with peanut, indicating a lack of mast cell degranulation, consistent with the lack of symptoms and stable vital signs.

Although peanut‐specific IgE was undetectable, and monkeys did not experience anaphylaxis upon OFC, we investigated whether there was any evidence of immunogenicity. To do so, we quantified peanut‐specific IgG throughout the course of peanut exposure via ELISA. Interestingly, seven of the eight monkeys produced increasing peanut‐specific IgG by day 32 (Figure 2C,D), with no significant differences between male and female monkeys (Figure 2C). When all monkeys were combined, the increase in peanut‐specific IgG at day 32 compared with the baseline reached statistical significance (*P* < .05). These results indicate that repeated exposure to peanut on the skin is immunogenic.

## 4 DISCUSSION

While monkeys did not become sensitized to peanut in the current model, several limitations could be addressed. Future studies should investigate longer periods of exposure (eg, 8 weeks instead of 4) and higher doses of peanut in a dose‐response manner. In addition, the application of peanut to a disrupted skin barrier has been demonstrated to promote sensitization in humans,^5^ which may also be required to sensitize primates. Interestingly, recent studies from the LEAP cohort demonstrated that *Staphylococcus aureus* colonization on the skin was associated with peanut allergy.^15^ Furthermore, the application site and relative thickness of the skin may be other important considerations for future studies. While the 1.2‐ to 1.4‐year‐old monkeys used in this study are similar in age to when children may develop a peanut allergy, the use of younger monkeys in future studies may enable sensitization. Notably, experiments using monkeys are costly, limited to small sample sizes, and restricted in terms of procedural capabilities compared with rodent studies, which limits the number of variables that can be investigated in a single experiment.

In summary, by adapting a similar protocol to that used in mice,^13^ we were unable to detect any peanut‐specific IgE production postcutaneous peanut exposure, indicating that this protocol did not sensitize the monkeys to peanut. Furthermore, monkeys challenged with 200 mg of peanut extract did not experience any allergic symptoms or detectable histamine release, consistent with a lack of sensitization. However, these monkeys produced increasing quantities of peanut‐specific IgG throughout the course of peanut exposure, indicating that the application of peanut through the skin is immunogenic. Ultimately, our data do not exclude that monkeys, or humans, can be sensitized by cutaneous exposure to peanut, we can only conclude that this particular protocol and study population did not become allergic.

## CONFLICT OF INTERESTS

The authors declare that there are no conflict of interests.

## Data Availability

The data that support the findings of this study are available from the corresponding author upon reasonable request.
